# Evaluating the Impact of a Clinician Improvement Program for Treating Patients with Intellectual and Developmental Disabilities: The Challenging Case of Mississippi

**DOI:** 10.3390/healthcare6010003

**Published:** 2018-01-10

**Authors:** John P. Bartkowski, Janelle Kohler, Craig L. Escude, Xiaohe Xu, Stephen Bartkowski

**Affiliations:** 1 Department of Sociology, The University of Texas at San Antonio, San Antonio, TX 78249, USA; xiaohe.xu@utsa.edu (X.X.); stephenbartkowski@gmail.com (S.B.); 2 Department of Psychology, The University of Texas at San Antonio, San Antonio, TX 78249, USA; janellekohler@gmail.com; 3 DETECT of Mississippi, 100 Hudspeth Center Drive, Highway 475 South, Whitfield, MS 39193, USA; clejxn@yahoo.com

**Keywords:** disabilities, intellectual disability, developmental disability, physician, nurse, clinician, medical, evaluation, deinstitutionalization, health disparities

## Abstract

In recent years, people with intellectual and developmental disabilities (IDD) have moved from institutionalized settings to local community residences. While deinstitutionalization has yielded quality of life improvements for people with IDD, this transition presents significant health-related challenges. Community clinicians have typically not been trained to provide sound medical care to people with IDD, a subpopulation that exhibits unique medical needs and significant health disparities. This study reports the results of a comprehensive evaluation of an IDD-focused clinician improvement program implemented throughout Mississippi. DETECT (Developmental Evaluation, Training and Consultative Team) was formed to equip Mississippi’s physicians and nurses to offer competent medical care to people with IDD living in community residences. Given the state’s pronounced health disparities and its clinician shortage, Mississippi offers a stringent test of program effectiveness. Results of objective survey indicators and subjective rating barometers administered before and after clinician educational seminars reveal robust statistically significant differences in clinician knowledge and self-assessed competence related to treating people with IDD. These results withstand controls for various confounding factors. Positive post-only results were also evident in a related program designed specifically for medical students. The study concludes by specifying a number of implications, including potential avenues for the wider dissemination of this program and promising directions for future research.

## 1. Introduction

The Centers for Disease Control and Prevention reports that about one in every five American adults—some 53 million citizens—has a disability of some sort [1], and recent evidence reveals that about one in eight (12.5%) has a disability that poses significant functional limitations [2]. Mississippi is among the states with the highest percentage of its residents living with disabilities [1]. There are different types of disabilities, with some featuring cognitive impairments (intellectual disabilities) and others consisting of physical or mobility limitations (developmental disabilities). Regardless of the specific form of disability, the health-related challenges faced by Americans with intellectual and developmental disabilities (IDD) are formidable. People with IDD are more likely to be impoverished and undereducated, have a reduced lifespan, and are significantly more susceptible to a host of health disparities that include overweight and obesity, substance use, and lack of emotional support, all of which are compounded by problems accessing adequate care [1,2,3,4]. In fact, the reduced lifespan and diminished quality of life faced by Americans with IDD rival—and, in many cases, exceed—the health disparities of other disadvantaged groups [2].

One primary cause of health disparities among people with IDD is the lack of sound and effective medical care available to this vulnerable subpopulation combined with these patients’ specialized medical needs [2,3,4,5,6,7,8]. The transition of many people with disabilities into non-institutionalized community settings has facilitated the personal and social flourishing of people with IDD [9], but poses serious challenges for community-based medical providers who generally lack training related to IDD-focused patient care. Medical clinician training programs designed to bolster care for patients with IDD have surfaced in a few locales, but remain relatively rare [10]. Some IDD-focused clinician training programs that have been examined and recommended as models have proven effective, in part, because they are filling an egregious gap in the field [2,10,11]. For example, incentivized clinician improvement programs have effectively encouraged medical providers to conduct more regular annual health checks of people with IDD [12]. Other research emphasizes the value of medical school courses that teach communication skills suited for patients with IDD [13]. However, overall, IDD-focused clinician improvement programs have exhibited mixed success. While a few programs have proven effective, several have failed to deliver anticipated results due, in part, to entrenched negative attitudes toward people with IDD among many medical professionals, particularly those that have little experience with disabilities [14,15,16]. The general paucity of such clinician improvement programs and the mixed results they have produced indicate that much remains to be learned about how to train clinicians to enhance their capacity to deliver effective care to patients with IDD.

The present study advances this nascent body of research by reporting evaluation results of a clinician improvement program designed to bolster Mississippi medical providers’ capacity to deliver care to people with IDD. The program was implemented by DETECT (Developmental Evaluation, Training and Consultative Team) of Mississippi. The evaluation focuses on objective knowledge transfer and subjective competence self-assessments evident among participating clinicians. These objective impacts were complemented by changes in seminar attendees’ subjective appraisals of their ability to treat patients with IDD effectively. This subjective component of the evaluation is important because clinicians’ confidence in their own ability to deliver effective care shapes their capacity to treat patients with IDD and their experiences doing so [10]. Additional evaluation activities ascertained subjective assessments of impact associated with DETECT’s IDD-X (short for “The IDD Experience”) program targeted at medical students. Using post-event surveys, descriptive statistical results buttress the evaluation findings that emerged from the clinician education seminars.

## 2. Program, Context, and Hypotheses

Given the formidable nature of IDD-related health disparities and the move toward home and community-based residences for people with IDD, DETECT (Developmental Evaluation, Training and Consultative Team) of Mississippi was established in November 2014 (http://detectms.com/). DETECT’s mandate involved addressing the knowledge and skill gaps associated with the provision of healthcare to people with IDD. Among its various programs, DETECT has provided education, training, consultation, informational resources, and referral services to clinicians with limited expertise in treating patients with IDD. One of the centerpiece programs offered by DETECT involved medical education seminars targeted at clinicians, including medical doctors, osteopathic physicians, nurse practitioners, dentists, and other providers of medical services.

DETECT’s clinician education seminars, each of which was typically about ninety minutes in duration, had a principal focus on diagnostic knowledge and treatment techniques germane to patients with IDD. Still, sustained attention to medical knowledge and skills was situated within a broader constellation of issues that bear significantly upon healthcare service delivery to people with IDD. The seminars began by discussing legal definitions and social trends associated with disabilities. Included in this portion of the presentation was a discussion of deinstitutionalization, that is, the movement of people with IDD from institutionalized settings to residential living. This material was deemed important because, apart from providing noteworthy contextual information, it underscored the need for capable community-based medical service provision to people with IDD. Next, the prevalence of IDD-related health conditions and the distinctive healthcare needs of patients with IDD were reviewed. Core content of the seminars involved a review of common medical conditions faced by people with IDD. This portion of the seminar provided in-depth coverage of the “fatal four” medical conditions (constipation, aspiration, dehydration, and seizures). These conditions are quite prevalent among people with IDD and, if unaddressed, can generate more complicated and severe medical problems. Specialized medical techniques that have proven effective in diagnosing and treating people with IDD also received considerable attention in the seminars. For example, because symptoms of disease are typically subtle and often difficult to ascertain among patients with IDD, techniques for creating, maintaining, and utilizing an accurate seizure record to treat seizures were discussed. To foster valuable professional networks with seminar attendees, presentations also addressed the locations of DETECT offices around Mississippi and ongoing consultative services provided by DETECT. At the conclusion of each seminar, professional contact information was collected from trained clinicians who were willing to be included in a provider network database that DETECT could enlist to refer patients while offering consultative support as desired. This provider network reflected an effort to improve the medical infrastructure in Mississippi. It is important to note that educational seminars (not referrals and consultations) are the subject of this evaluation.

In 2015, an evaluation of DETECT of Mississippi services was developed by the first author in consultation with DETECT’s clinical director (third author). Additional collaboration was undertaken with evaluation team members (second, fourth, and fifth authors). The core elements of DETECT’s educational seminars were eventually integrated into a related program (IDD-X) specifically targeted at medical students. While the content of IDD-X was similar to that provided in the seminars for established professionals, IDD-X aimed to provide knowledge and model select treatment techniques for those in the professional pipeline. Evaluation results from the DETECT seminars are the central focus of this evaluation, but select results are also reported from IDD-X. Overall, this study demonstrates how pretest/post-test objective and subjective evaluation methods can be used (DETECT clinician seminars) while also addressing how a post-only evaluation of subjective impact can be implemented (IDD-X). Therefore, this study reports a variety of results related to an IDD-focused clinician improvement program while illustrating diverse evaluation methods for assessing such initiatives.

For several reasons, Mississippi provides a stringent test case for a clinician improvement program that aims to enhance care provided to patients with IDD. First, Mississippi is among the states with the highest proportion of citizens with disabilities [1]. The vast majority of medical professionals served by DETECT have had considerable prior experience treating patients with IDD but have had little or no training to treat such patients. Second, Mississippians at large and those with disabilities in particular face a number of contextual challenges, including entrenched poverty, persistent racial-ethnic segregation, rural remoteness and, most relevant to this program, severe mortality and morbidity rates [1,17,18,19]. Third, Mississippi faces a healthcare provider shortage, with many communities throughout the state defined as medically underserved [20]. Given these contextual factors and the steep healthcare demands faced by Mississippi medical providers, an effective medical provider education program for Mississippi clinicians is likely to be successful in states presenting less formidable challenges. Hence, opportunities for wider dissemination are quite promising.

Evaluation services were provided from 2015 to 2017 at a competitive market rate, and the research team adopted a participatory, utilization-focused approach to evaluation [21]. In preparation for the evaluation, the research team analyzed a number of items from the 2013 to 2014 National Core Indicators (NCI) data (report available from first author by request). This assessment revealed that medical service gaps are most common among Mississippians with mild or moderate disabilities. For example, while 90.6% of Mississippians with a severe (that is, profound) disability had an annual physical exam in the past year, only 73.1% of Mississippians with a mild disability and only 73.7% of those with a moderate disability had a physical exam within one year of the survey. Although these statistical differences are noteworthy, their social relevance is even more striking. Mississippians with a mild or moderate disability are those who are most likely to live in community settings. In addition, if data from the NCI sample are to be used as a guide to the distribution of disabilities within the state, Mississippians with a mild disability or a moderate disability far outnumber those with a severe disability. Among the 335 Mississippians with disabilities in the NCI sample for whom last annual physical exam data were available, those with a mild disability (*n* = 186; 56% of full sample) or a moderate disability (*n* = 99; 30% of full sample) significantly outnumbered their peers with a severe or profound disability (*n* = 32; 10% of full sample). (The remaining 4% of NCI respondents composed a residual unclassified category.) Thus, available data underscored the need for a program like DETECT, which aims to train clinicians throughout the state to provide effective care to Mississippians with disabilities living in community residences.

This study aims to examine the effectiveness of DETECT’s educational seminars for medical providers and medical students. Given the foregoing contextual factors in Mississippi and the pressing need for IDD-focused clinician education programs, the study is governed by the following hypotheses.

- H1: Significant gains (*p* < 0.05) in clinician knowledge about IDD and related factors will be observed when objective scores of pre-seminar and post-seminar knowledge are compared.
- H2: Significant improvement (*p* < 0.05) in clinician self-assessments of IDD treatment competence will be observed when pre-seminar and post-seminar competence barometer scores are compared.
- H3: At least 75% of medical student trainees who complete IDD-X will evaluate this program using the most positive (superlative) rating option available on post-only training impact assessments.

## 3. Methods

All evaluation instruments were developed by the first author and were composed of proprietary quality assurance measures developed to align with the educational content of the seminars. Possible validity threats were examined through a two-phase instrument validation process that included (1) an internal review procedure whereby DETECT personnel assessed proposed evaluation items to ensure correspondence with training content and (2) an external review procedure whereby the proposed instrument was field-tested among seminar attendees and assessment-related feedback was solicited from attendees through a facilitated discussion. Only one item (DETECT clinic location response options) was slightly altered through the review procedure. Survey data related to the instrument validation process were excluded from the actual program evaluation because such data were used only for validation purposes. Having ascertained no meaningful threats to measurement validity, the indicators were integrated into final evaluation instruments (see Table 1). Prior to the collection of quality assurance data, all program participants were apprised of their rights pertaining to the evaluation. These considerations included the purpose of the evaluation, the voluntary nature of their participation in the evaluation, and their rights pertaining to the extent of their participation (ability to skip any questions or refuse to participate altogether). As is common with program evaluation, no formal Institutional Review Board approval was required because evaluation activities consist of program monitoring and quality assurance activities that do not conform to the federal definition of research.

Data for this study are drawn from two instruments. First, an educational seminar pretest/post-test survey was developed and administered to participating clinicians to ascertain demographic characteristics, professional factors (e.g., field, experience), knowledge transfer, and self-assessments of treatment competence related to patients with IDD. Knowledge quiz data were collected from 544 seminar attendees who completed those pretest and post-test survey items. Descriptive statistical data on these respondents are featured in Table 2 and are discussed in detail in Section 4.1. Fewer seminar attendees (*n* = 447) completed the treatment competence barometer. The competence barometer was targeted at clinicians actively treating patients. Therefore, healthcare administrators or others that did not see patients understandably opted out of responding to the competence barometer. Women, Caucasians, and physicians were the most common respondent types for both of these measures. Power analyses for each of these study samples were completed and demonstrated that study sample sizes were sufficient to conduct meaningful statistical analyses. (Results of power analyses are available by request from the first author.) Second, a post-event survey was developed and administered to IDD-X participants (159 medical students) to gauge participant ratings pertaining to satisfaction, effectiveness, and the projected impact of these events. Sociodemographic data were not collected from IDD-X participants in an effort to keep the post-event survey concise (one single page) and to permit the acquisition of qualitative improvement feedback. The qualitative feedback was analyzed separately from the results presented in this study and is not featured in this investigation. Evaluation participation rates in both of these programs were greater than 90%. This participation threshold minimizes threats to results that might otherwise be posed by selectivity bias due to evaluation refusals. All data used for the pretest/post-test portion of the evaluation were based on matched survey responses. The pretest and post-test surveys were duplexed onto opposite sides of the same sheet of paper to facilitate matching.

### 3.1. Dependent Variables

Dependent Variable 1: Performance on Objective Knowledge Quiz (Pretest versus Post-Test). This study uses a series of dependent (outcome) variables to examine the impact of attendance at DETECT training seminars designed to teach medical professionals critical knowledge about medical diagnosis, treatment, and related capacities associated with serving patients with IDD. The first dependent variable consists of correct answers to a six-item knowledge quiz administered before and immediately after each training seminar. Core measures of this knowledge quiz are featured in Table 1, with the correct response for each item presented in bold italicized text. When circumstances warranted, a few alternative quiz items were developed to account for different attendee audiences and seminar subject matter. For example, dental knowledge questions replaced several of the physician knowledge questions when the seminar presentation was adapted for dentist attendees. Given the periodic use of alternative measures, the first dependent variable examines composite scores of correct responses at pretest and post-test rather than focusing on individual items. Alternative measures are available from the first author by request, as are analyses of individual items at pretest versus post-test.

The first core quiz item examined knowledge of an important contextual factor related to the living arrangements of people with IDD. Quiz item 1 inquired about trends in living arrangements for persons with disabilities, with the correct response being community living. Quiz items 2–5 tested clinician knowledge of medical needs and treatment strategies related to patients with IDD. These items were developed from training seminar content that was itself based on extensive reviews of published studies on the health, medical needs, and treatment options for patients with IDD. Item 2 inquired about the “fatal four” conditions for people with IDD. The conditions to which the fatal four refer are constipation, aspiration, dehydration, and seizures. These conditions, along with their relation to IDD health and treatment strategies, were addressed in the training. Because item 2 asks attendees to identify which of the presented responses is not one of the fatal four, the correct response is muscle atrophy. Knowledge quiz item 3 asked seminar attendees about an action that can be employed to prevent aspiration among persons with disabilities, with the correct response being position body properly. Knowledge quiz item 4 asked seminar attendees about an effective effort for managing seizures among persons with IDD. The last response option, namely, reliance on an accurate seizure record, is the correct response. General practitioners and family physicians need to be attentive to the overall health of their patients. Among those with IDD, general problems associated with dental health can be common. For that reason, seminar attendees were asked about a common dental issue faced by persons with IDD. Teeth grinding is the correct response. These items were included in the core knowledge quiz because they were all addressed in detail as part of the standard training sessions delivered to clinicians.

In addition to these five core knowledge quiz measures, item 6 asked attendees about the locations of DETECT offices in the state of Mississippi. This question is important because seminar trainees were invited to develop consultative and, as needed, referral relationships with the DETECT office nearest to them. Post-training surveys also inquired about trainees’ willingness to consult with DETECT related to treating patients with IDD or, as circumstances permitted, to accept patient referrals from DETECT with support from DETECT as needed. The vast majority of trainees readily agreed to these consultative and referral relationships on the post-test. However, such collaborative ties would be less likely to materialize if the trainees were not aware of DETECT locations in the state of Mississippi. Therefore, trainees were asked to identify DETECT’s three locations in the state by selecting the community in which DETECT does not have a clinic. The correct response, the community without a DETECT office, is Southaven.

Overall, then, these knowledge quiz items test trainees’ broad-based understanding of common living circumstances of people with IDD (quiz item 1), medical needs and treatment considerations typically evident for patients with IDD (quiz items 2–5), and DETECT locations for ongoing consultative support and referrals (quiz item 6). To construct this dependent variable, the number of correct responses, respectively, before and after seminar completion were secured and summed across all responding attendees. In this way, a composite score of correct responses at pretest could be compared with a composite score of correct responses at post-test. The difference between these two composite scores was then used to render a magnitude of change measure with respect to participant knowledge. Because correct responses at post-test exceed correct responses at pretest, this dependent variable can be treated as a measure of knowledge gain.

Dependent Variable 2: Subjective Assessments of Treatment Competence (Pretest versus Post-Test). A second dependent variable associated with seminar trainee attendance is a single item subjective measure. This measure is designed to examine pre-seminar to post-seminar improvements in clinician self-ratings of their competence to treat a patient with IDD. Because these ratings are rendered through the trainees’ self-assessment on a 100-point scale, this measure is called a subjective competence barometer. This competence barometer is measured through the following question: “On a scale from 1 to 100 where 100 indicates a maximum ability to deliver effective medical care to people with IDD, how would you rate your ability to provide such care?” A blank write-in space was presented to each seminar attendee with a prompt to “indicate [a] number from 1 to 100.” These responses were then coded to reflect the actual numerical rating provided by the seminar attendee and constituted a continuous measure.

To create this dependent variable, all competence barometer scores provided by seminar attendees at pretest were summed and divided by the number of scores to arrive at a mean pretest competence rating. The same procedures were then used to generate a mean post-test competence rating for all seminar attendees. Recall that for both this dependent variable and the foregoing dependent variable, magnitude of change from pretest to post-test is the key point of interest. Therefore, the difference between these subjective ratings (barometer self-assessment scores) serves as the second dependent variable. A greater average rating at post-test is indicative of an increase in subjectively assessed treatment competence among seminar attendees. In this way, treatment competence improvement is the second dependent variable.

Dependent Variable 3: IDD-X Subjective Impact Ratings (Post-Test Only). A final set of outcome measures is related to IDD-X, a program that is quite similar to the training seminars while offering a more intensive immersive experience through on-site clinic modeling of treatment techniques in addition to knowledge-based training. These subjective post-only measures are featured in this study because they represent a more concise and economical approach to clinician improvement program evaluation. They can be useful when evaluation funds are scarce or nonexistent because they can be simply secured, easily entered into a data management system, and quickly tallied to generate overall trainee appraisals. Because these outcomes are ascertained at post-test only, the results for these items are featured in this study as descriptive statistics. Thus, regression models are not suitable for these measures.

The seven post-only IDD-X measures and response options are as follows: (1) Overall, how satisfied are you with IDD-X? (a) Very dissatisfied, (b) Somewhat dissatisfied, (c) Somewhat satisfied, or (d) Very satisfied; (2) How effective was the presenter in communicating medical information about people with disabilities? (a) Very ineffective, (b) Somewhat ineffective, (c) Somewhat effective, or (d) Very effective; (3) I would recommend IDD-X to a colleague of mine; (a) Strongly disagree, (b) Somewhat disagree, (c) Somewhat agree, or (d) Strongly agree. The response categories for items 4–7 are the same as those featured in item 3 (four-point Likert scale measuring extent of agreement): (4) IDD-X increased my awareness of the special medical needs of people with disabilities; (5) IDD-X will change the way I treat patients with intellectual and developmental disabilities; (6) I would consider using DETECT for referrals or consultations when treating a patient with IDD; and (7) Medical students would benefit from having additional IDD healthcare training while in medical school. As noted, these data are reported in descriptive form for each item as they were secured from IDD-X attendees. For ease of presentation in this study, the lower two rating categories (e.g., strongly dissatisfied and somewhat dissatisfied) were collapsed into a single category. Distinctions between the two positive rating categories were preserved in this study in the same way they were initially presented to attendees on the survey. Preserving these positive rating categories permits proportions of the superlative rating thresholds (most positive category) to be compared across measures.

### 3.2. Control Variables

One of the benefits of using regression models to analyze change for the first two dependent variables is the ability to include control variables in statistical models. The inclusion of control variables permits researchers to account for the potential influence of confounding factors that might alter the relationships between the dependent variables (outcomes) and independent variables (predictors). The following factors were ascertained on baseline (pretest) surveys and are controlled in regression models for the first two dependent variables (that is, objective quiz knowledge and subjective assessments of treatment competence). (These controls are not relevant to the third dependent variable because those ratings are reported as descriptive data only.)

Turning to the specific control variables, professional experience was ascertained as the number of years practicing in the profession. This control variable is included because more years of experience could provide a greater number of opportunities to treat patients with IDD. Trainees were also asked to indicate their gender (male or female) and their race-ethnicity (white, African American, other) because it is possible that trainings would differ in their effectiveness for men and women clinicians or for clinicians of different racial-ethnic backgrounds. Finally, occupation (physician, nurse, other) was ascertained to examine the relative effectiveness of the seminars for those with different roles in the field of medicine. Various types of physicians (e.g., medical doctors, osteopaths) and nurses (e.g., nurse practitioners, registered nurses) were served, but no discernible differences among these more specific subgroups were evident, so they were combined into broader categories for ease of presentation.

### 3.3. Analytical Strategies

Random effects regression analyses were conducted for the first two dependent variables. Regression analyses of the first dependent variable—that is, objective knowledge gains—were conducted in two phases. First, a regression model was generated to determine zero-order (unmediated) relationships between pretest correct responses compared with post-test correct responses. Second, after zero-order relationships between pretest and post-test correct responses were determined, controls were added to a second regression model to determine if the inclusion of these covariates attenuated (reduced or eliminated) any significant findings initially evident in the zero-order relationship. It should be noted that correct responses for all items at each respective time point were combined to measure gross knowledge gains from pretest to post-test. Individual item analyses were conducted separately and are not presented here. The results associated with analyses of individual measures are available by request. The regression models focused on the first dependent variable (objective knowledge gains) and were used to test Hypothesis 1, which predicts an established pretest to post-test threshold gain (*p* < 0.05).

A similar two-phase series of random effects regression models were also conducted for the second dependent variable, namely, the treatment competence barometer. Aggregated pretest ratings (scores from 1 to 100) were compared with aggregated post-test ratings (again, on a scale from 1 to 100) in a series of two random effects regression models. In the first model, the zero-order relationship was ascertained between pretest ratings (combined from all attendees) and post-test ratings (again, aggregated among all attendees) without regard for any possibly confounding factors. Thus, this zero-order model features no control variables. The second random effects regression model introduces all of the control variables described above to determine if the zero-order relationship between pretest and post-test competence ratings in the baseline regression model is attenuated by the inclusion of covariates. These models for the second dependent variable (treatment competence improvements) are used to test Hypothesis 2, which predicts a pretest to post-test threshold gain akin to that for the first hypothesis (*p* < 0.05).

Regression was not needed for the third dependent variable, namely, subjective impacts of IDD-X. Because that third dependent variable is composed solely of raw categorical data from post-only surveys, descriptive statistics were sufficient to determine degrees of satisfaction and effectiveness. Actual degrees of satisfaction and effectiveness are scrutinized relative to thresholds specified in Hypothesis 3. Thresholds featured in Hypothesis 3 specify a minimum of 75% of attendees selecting the superlative (most positive) rating category.

## 4. Results

Having described the methods that were employed to evaluate DETECT’s educational seminars, we now turn to examining the results of the evaluation. First, descriptive statistics are presented for each of the two outcome measures that were the focus of the clinician seminar evaluation: (1) change in the aggregated number of correct responses on the series of pretest/post-test knowledge quiz items administered to seminar attendees; and (2) change in medical providers’ self-rated competence for treating people with IDD effectively before and after the seminars.

### 4.1. Descriptive Statistical Findings

Table 2 presents descriptive statistics related to each of the first two outcome measures, along with key characteristics of seminar attendees. Turning first to the aggregated number of correct responses on the pretest/post-test knowledge quiz, data are available from 544 seminar attendees. As indicated by the top portion of the left-most numerical column featured in Table 2, the mean of correct pretest responses is 3.45 out of six questions posed to attendees. By contrast, the mean of correct post-test responses is 4.72. The second numerical column in Table 2 features the standard deviations for these respective distributions. (Rigorous tests of statistical difference between these pretest and post-test results are not presented until Table 3.) Additional data on attendees who responded to the knowledge quiz items are featured in the left-most column as time-invariant controls, wherein raw numbers are presented in the left-most column and percentages are featured in the adjacent column to the right. The typical seminar attendee had about seventeen years in the field. A greater percentage of women (64%) attended the seminars than men (36%) and just under three-quarters of attendees were white (72%). Physicians (47%) composed the largest proportion of seminar trainees, while roughly one in five trainees were nurses (20%). About one third of trainees (33%) worked in the healthcare field, but were not physicians or nurses. Many of these were technically trained (e.g., physician’s assistants), while others were direct service providers (sometimes called direct support professionals) for whom DETECT seminars yielded valuable knowledge.

Table 2 also presents data on the seminar attendees’ self-rated ability to provide care to patients with IDD. Recall that this treatment competence barometer is measured as a subjective self-appraisal offered by seminar attendees at pretest (before seminar attendance) and at post-test (after seminar attendance). Mindful that the possible range of this treatment competence barometer is 1–100, the average self-appraisal at pretest is 49.25 while the average post-test self-appraisal is 66.75. Standard deviations for these two mean measures are presented in the right-most column. The difference between these subjective rating mean scores is 17.5 raw points, which constitutes a 35.5% increase in average subjective competence ratings from pretest to post-test. There is some sample attrition across dependent variables, such that more attendees provided knowledge quiz responses at pretest and post-test (*n* = 544) than subjective treatment competence ratings (*n* = 447). Nevertheless, these two groups of attendees exhibit rough similarity in terms of years of experience, gender, race, and occupation. Consequently, while there is some case loss for the second of the two dependent variables, the subsamples exhibit comparable key attributes.

### 4.2. Results of Regression Analyses

Descriptive statistics provide a useful overview of general patterns exhibited in the data. However, they cannot examine the relative associations between predictor and outcome variables net of controls for possible confounding factors. Random effects regression models were used to examine the degree of association between key variables—namely, pretest versus post-test scores—in a zero-order fashion (no control variables) and net of controls for possible confounding factors.

Table 3 presents the results of random effects regression models that were used to analyze changes in correct answers on the pretest/post-test knowledge quiz administered to seminar attendees. Table 4 features the results of another series of random effects regression models that were conducted to analyze changes in subjective treatment competence barometers from pretest to post-test. The results of two different regression models are featured in Table 2 and Table 3. In Model 1, the zero-order difference between pretest and post-test scores is examined without any control variables. If a positive beta coefficient is statistically significant in Model 1, that finding would indicate a meaningful gain in knowledge (Table 3) or self-rated treatment competence (Table 4) for seminar attendees from pretest (before the seminar) to post-test (after the seminar). While Model 1 may indicate positive changes among seminar attendees, a more rigorous test is introduced through Model 2. In Model 2, control variables are integrated into the regression equation to determine if the inclusion of those additional variables attenuates (that is, reduces or eliminates) a significant zero-order relationship that might have surfaced in Model 1. If no such attenuation is found in Model 2, then positive statistically significant results would be evident across attendees who differ in terms of years of experience, gender (men and women), race-ethnicity (white, African Americans, and other), and provider type (physician, nurse, and other).

Recall that Hypothesis 1 anticipates a significant difference (*p* < 0.05) in objective knowledge gains from pretest to post-test for DETECT clinician training seminar attendees. This hypothesis is tested in Models 1 and 2 of Table 3. Model 1 reveals that, when compared with pretest knowledge scores, the zero-order coefficient for post-test knowledge is positive and highly significant (*p* < 0.001). This finding indicates that seminar attendees were significantly more likely to select a correct knowledge quiz answer at post-test when compared with pretest. Ancillary analyses (not shown here) revealed significant results across a number of different measure types. Therefore, for various waves of seminar attendees situated in different community locales across Mississippi, significant knowledge gains were exhibited concerning the movement of people with IDD into community residences (quiz item 1), health challenges and appropriate treatment techniques for patients with IDD (quiz items 2–5), and DETECT locations for ongoing consultative support (quiz item 6). In short, seminar attendees exhibited broad-based knowledge gains.

Model 2 of Table 3 reveals that these statistically significant knowledge gains from pretest to post-test withstand the inclusion of various control variables because the significance level is not diminished from Model 1 (*p* < 0.001) to Model 2 (*p* < 0.001). Therefore, confidence in the results is warranted for attendees regardless of years of experience, gender, race-ethnicity, and provider type. The only exception to this pattern is that African American attendees were somewhat less likely to evince pretest to post-test knowledge gains than were those from a racial-ethnic background identified as other (non-white, non-black). Hypothesis 1 is strongly supported for both the zero-order relationship between pretest and post-test scores (no controls) (Model 1) and, again, for pretest to post-test knowledge changes net of confounding factors (Model 2).

Table 4 examines the second dependent variable analyzed in this study, namely, changes in the subjective self-appraisal of treatment competence on a scale from 1 to 100. Zero-order changes from pretest to post-test in this competence barometer are examined in Model 1 of Table 4 without consideration of control variables. Hypothesis 2 anticipated significant competence barometer changes (*p* < 0.05) from pretest to post-test. Subjective self-appraisals of treatment competence increased over the course of DETECT seminars in a manner that meets the highest threshold of statistical significance (*p* < 0.001). These results persist in Model 2 despite the inclusion of controls that could reasonably be expected to attenuate the magnitude of change in pretest to post-test self-appraisals. The only significant control variable is physician, which indicates that physicians were more likely to exhibit competence barometer gains than those who self-identified with the other occupational category. In ancillary analyses, the change scores of subjective treatment competence ratings were compared for individual seminars. With remarkable consistency, changes in barometer scores were highly significant across seminars (*p* < 0.001). Hypothesis 2 is strongly supported by these results. In summary, for both the first and the second dependent variables, change scores from pretest to post-test eclipse the highest threshold of statistical significance and these results are sustained even when confounding factors are considered through the introduction of various control variables.

### 4.3. Subjective Impact of DETECT’s IDD-X Program for Medical Students

Much of the same training content featured in the seminars was also addressed in a related DETECT program called IDD-X. However, IDD-X (short for “The IDD Experience”) offered more intensive exposure to the unique needs of patients with IDD and, when possible, modeled particular treatment techniques at DETECT’s clinic. IDD-X was targeted at changing the capabilities of those in the clinician pipeline, namely, medical students. Pretest/post-test scores (featured above) had already validated the effectiveness of the training content and delivery to medical providers at large and were, therefore, not used to evaluate IDD-X. Rather, IDD-X used a post-only survey to examine the perceived impact of these training sessions. Consequently, the evaluation of DETECT’s IDD-X program models a series of useful measures to employ when post-only evaluation designs are the most feasible option for evaluating an IDD-focused clinician training program. Post-only evaluation designs are more economical and less data-intensive than pretest/post-test designs.

Recall that Hypothesis 3 anticipated IDD-X ratings wherein at least 75% of medical student trainees would select the superlative (highest) rating category for each of seven measures. Table 5 complements findings reported thus far by illustrating how an IDD-focused medical training program can be evaluated efficiently with a post-only survey featuring a range of subjective impact measures. Given space constraints and limited evidence of negative ratings, the “very” and “somewhat” negative rating categories for each item, while appearing separately in the survey, were collapsed into a single category for inclusion in Table 5. Table 5 reveals strongly positive participant ratings of IDD-X. The maximum number of responses was 159, and the highest rating category captured the vast majority of participant responses, ranging from 88% for item 1 (overall satisfaction) to 96% for item 2 (presenter effectiveness). Nearly 94% of IDD-X participants reported increased awareness of the special medical needs of people with IDD and 89% stated that IDD-X would change their treatment approach. Roughly nine in ten participants would consider using DETECT for referrals or consultations (93%) and expressed strong agreement that medical students could benefit from more IDD-related training (91%). Each one of these findings provides robust support for Hypothesis 3.

## 5. Discussion

This study reports the results of an evaluation of medical provider and medical student training programs conducted by DETECT of Mississippi. These programs were organized around similar aims—namely, improve clinician knowledge and skills for treating patients with intellectual and developmental disabilities (IDD)—but were evaluated using different means. Clinician seminars were evaluated with pretest and post-test instruments to discern changes in objective knowledge and subjective competence before and after the seminars. IDD-X (that is, “The IDD Experience”) was targeted at medical students and offered onsite education and training while being evaluated through a post-test-only survey. As a medically underserved state featuring a high proportion of residents with IDD, Mississippi provides a stringent test of the effectiveness of the programs offered by DETECT. In short, if these programs prove to be effective in light of the myriad challenges posed by Mississippi’s social context, they are likely to be efficacious virtually anywhere.

The evaluation results reported here were unambiguously positive. By every means used to investigate their impact, DETECT seminars and IDD-X met a high threshold of success. Composite scores on objective knowledge quizzes administered before and after the DETECT clinician seminars exhibited highly significant changes (*p* < 0.001), even when a series of potentially confounding factors were controlled. In addition, ancillary analyses revealed clinician knowledge gains for many of the individual items on pretest/post-test surveys. Similar changes (*p* < 0.001) were evident when clinicians were asked to rate their own competence to treat patients with IDD before and after the DETECT seminars. Once again, this threshold of significance was not altered by the inclusion of control variables. Subjective self-appraisals of treatment competence at post-test (mean of 67 on a 1–100 scale) compared quite favorably to its counterpart pretest mean (49 on the 1–100 scale). IDD-X was rated with impact measures on a post-only survey, but far eclipsed the hypothesized minimum of 75% of trainees selecting the superlative (most positive) category for each rating domain. For the majority of domains, more than 90% of trainees selected the superlative rating category.

There are two clear implications associated with this study. First, there would seem to be great promise in disseminating DETECT programs to other states. As noted in the literature review of this study, there are few IDD-focused clinician training programs like those offered in Mississippi even though the need for such programs nationwide is profound. Therefore, the evidence amassed here underscores the value of increasing the scale and reach of this program or ones modeled after it. Second, as such clinician education programs are implemented in other locales, evaluations of them should continue in earnest. Quality improvement entails ensuring fidelity in the dissemination of programs into new locales by different service providers (clinician trainers). The measures developed for evaluating DETECT could be used in other sites in an effort to determine if the positive results observed in Mississippi can be replicated elsewhere. As such efforts are undertaken, additional measures and methods could be employed to evaluate an even wider range of the educational information shared with clinicians.

Several directions for future research present themselves in the wake of this study. Perhaps most notably, a compelling argument could be made that the litmus test of clinician education effectiveness is patient outcomes and, most ambitiously, significant reductions in aggregate IDD-related health disparities. Patient outcomes can be measured in a number of ways, including satisfaction with medical services received and health improvements (e.g., morbidity reductions). This evaluation of DETECT services was focused on clinicians, not patients. However, as IDD-focused clinician education programs proliferate and are evaluated, efforts to examine impacts on patients should be pursued. Of course, reductions in health disparities evident between patients with IDD and their counterparts without IDD, or among patients with distinctive levels of IDD severity, would take considerable time to determine, but is a worthwhile long-term objective.

There is also an argument to make for the utilization of additional methods in the evaluation of clinician education programs as assessment efforts with such programs proceed. First, this study utilized pretest and post-test surveys to discern knowledge gains and improvements in subjective treatment competence. Future evaluations could be complemented by the use of a follow-up (post-post-test) survey administered approximately three to six months after seminar completion. Follow-up surveys present logistical challenges (e.g., greater cost, respondent attrition), but they could illuminate the degree to which knowledge and treatment competence are retained with the passage of time. There is reason to believe that clinicians trained by DETECT retained the knowledge given the salience of the information presented to them. However, efforts to document such retention are warranted. Second, qualitative evaluation methods could be equally illuminating. Ethnographic fieldwork in clinics designed to treat patients with IDD might discern best practices and cautionary lessons evident from the careful observation of medical service provision in action. One-on-one or focus group interviews with clinicians might also highlight the most challenging experiences encountered by clinicians and the most effective educational techniques used in seminars. It is worth noting that DETECT evaluation surveys did include qualitative components and, for the most part, qualitative feedback to open-ended questions was very positive. Such data were not included in this study given the focus on statistical impacts. Yet, qualitative data can be a valuable quality improvement resource.

## 6. Conclusions

Given the prevalence of intellectual and developmental disabilities (IDD) in the United States as well as the health disparities faced by Americans with IDD, there is a compelling need for programs that train medical providers to deliver care to people within this important yet vulnerable subpopulation. The recent transition of people with IDD into community residences underscores the need for physicians, nurses, and other healthcare providers to be trained in the delivery of effective care to patients with IDD. The evaluation results presented here indicate that the potential for clinician education programs to yield significant impacts is robust. In fact, DETECT could be considered a model program that is ripe for dissemination to other locales. This program may be especially effective in other Southern states, given that many of them face challenges similar to those found in Mississippi (high proportion of the population with IDD, elevated poverty rates, population dispersion, and medically underserved localities). As the nation considers how best to serve the medical needs of residents with IDD, much can be learned from utilizing the strategies that have been successfully employed in Mississippi. Although Mississippi is sometimes known for leading the nation in negative social indicators, the effective IDD-related healthcare training efforts underway there demonstrate that this state is capable of providing leadership in a vitally important and challenging domain of healthcare service delivery.

## Figures and Tables

**Table 1:** Core Measures for Objective Knowledge Quiz Featured on Pretest/Post-Test Surveys.

1. Federal law supports which of the following trends in living arrangements for persons with disabilities? (a) family residence; (b) ***community living***; (c) dyadic living; (d) institutional residence
2. Which is not one of the “fatal four” for people with intellectual or developmental disabilities (IDD)? (a) constipation; (b) seizures; (c) ***muscle atrophy***; (d) dehydration
3. Which action can be employed to prevent aspiration among persons with disabilities? (a) reduce salt intake; (b) ***position body properly***; (c) suppress appetite; (d) dilute liquids
4. Which of the following is very helpful in managing seizures among persons with disabilities? (a) food diary review; (b) ophthalmology consult; (c) pain relief; (d) ***accurate seizure record***
5. Which one of these is a common dental issue faced by persons with disabilities? (a) gum recession; (b) jaw misalignment; (c) ***teeth grinding***; (d) halitosis
6. Which of these is not one of the three locations of a DETECT clinic in Mississippi? (a) ***Southaven***; (b) Whitfield; (c) Ellisville; (d) Oxford

Note: The correct response for each item is presented in bold italicized text.

**Table 2:** Developmental Evaluation, Training and Consultative Team (DETECT) Seminar Attendee Descriptive Statistics.

Variables	Pretest/Post-Test Knowledge Quiz (Number of Correct Answers) (*n* = 544)		Pre-Post Self-Rated Treatment Competence (Barometer 1–100) (*n* = 447)	
	Mean/*n*	SD/%	Mean/*n*	SD/%
*Pre-seminar*				
Correct Quiz Answers	3.45	1.43	-	-
Treatment Competence	-	-	49.25	25.31
*Post-seminar*				
Correct Quiz Answers	4.72	1.82	-	-
Treatment Competence	-	-	66.75	24.07
*Time-invariant Controls*				
Years of Experience	17.31	14.08	17.17	14.15
Gender				
Male	196	36.0	168	37.6
Female	348	64.0	279	62.4
Race				
White	394	72.4	336	75.2
African American	106	19.5	72	16.1
Other	44	8.1	39	8.7
Occupation				
Physician	258	47.4	217	48.5
Nurse	106	19.5	85	19.0
Other	180	33.1	145	32.5

**Table 3:** Random Effects Regression Models for Knowledge Quiz Gains.

Variables	Number of Correct Answers (*n* = 544)					
	Model 1			Model 2		
	b	95% CI	sig.	b	95% CI	sig.
Post-test	1.265	1.014–1.516	***	1.265	1.014–1.516	***
Years of Experience				−0.007	−0.019–0.005	
Female				−0.120	−0.485–0.244	
White				0.069	−0.464–0.602	
African American				−0.657	−1.272–0.043	*
Physician				0.424	−0.012–0.859	
Nurse				0.182	−0.260–0.625	
Constant	3.452	3.257–3.647	***	3.492	2.833–4.150	***
Wald X^2^	97.7		***	127.9		***
*n*	544			544		

Note: * *p* < 0.5; ** *p* < 0.01; *** *p* < 0.001.

**Table 4:** Random Effects Regression Models for Treatment Competence Improvement.

Variables	Subjective Rating of Treatment Competence (*n* = 447)					
	Model 1			Model 2		
	b	95% CI	sig.	b	95% CI	sig.
Post-test	17.860	15.439–20.280	***	17.914	15.497–20.331	***
Years of Experience				0.074	0.156–0.303	
Female				1.686	−5.731–9.104	
White				1.635	−8.588–11.858	
African American				7.585	−4.537–19.706	
Physician				18.437	9.763–27.112	***
Nurse				8.058	−0.627–16.742	
Constant	48.908	45.748–52.068	***	33.690	20.902–46.478	***
Wald X^2^	209.1		***	241.0		***
*n*	447			447		

Note: * *p* < 0.5; ** *p* < 0.01; *** *p* < 0.001.

**Table 5:** Subjective Impact Ratings of DETECT’s IDD-X Program.

Survey Items and Response Options	Frequency	Percent
**1. Overall, how satisfied are you with IDD-X?**		
Very or somewhat dissatisfied	2	1.2
Somewhat satisfied	17	10.7
Very satisfied	140	88.1
**2. How effective was the presenter in communicating medical information about people with disabilities?**		
Very or somewhat ineffective	1	0.6
Somewhat effective	6	3.8
Very effective	152	95.6
**3. I would recommend IDD-X to a colleague of mine.**		
Strongly or somewhat disagree	3	1.9
Somewhat agree	12	7.5
Strongly agree	144	90.6
**4. IDD-X increased my awareness of the special medical needs of people with disabilities.**		
Strongly or somewhat disagree	0	0
Somewhat agree	10	6.3
Strongly agree	149	93.7
**5. IDD-X will change the way I treat patients with intellectual and developmental disabilities.**		
Strongly or somewhat disagree	1	0.6
Somewhat agree	16	10.1
Strongly agree	141	89.2
**6. I would consider using DETECT for referrals or consultations when treating a patient with IDD.**		
Strongly or somewhat disagree	0	0
Somewhat agree	11	6.9
Strongly agree	148	93.1
**7. Medical students would benefit from having additional IDD healthcare training while in medical school.**		
Strongly or somewhat disagree	2	1.3
Somewhat agree	13	8.2
Strongly agree	144	90.6
